# Surface tension coupled non-uniformly imposed flows modulate the activity of reproducing chemotactic bacteria in porous media

**DOI:** 10.1038/s41598-023-31753-y

**Published:** 2023-04-05

**Authors:** William Kuipou, Alidou Mohamadou

**Affiliations:** 1 African Centre for Advanced Studies, P.O. Box 4477, Yaoundé, Cameroon; 2 Centre for Research in Infectious Disease, P.O. Box 13591, Yaoundé, Cameroon; 3 National Advanced School of Engineering of Maroua, University of Maroua, P.O. Box 46, Maroua, Cameroon

**Keywords:** Biological physics, Biophysics

## Abstract

This paper investigates a non-homogeneous two-dimensional model for reproducing chemotactic bacteria, immersed in a porous medium that experiences non-uniformly imposed flows. It is shown that independently of the form of the fluid velocity field, the compressible/incompressible nature of the fluid significantly shifts the Turing stability-instability transition line. In dry media, Gaussian perturbations travel faster than the hyperbolic secant ones, yet the latter exhibit better stability properties. The system becomes highly unstable under strong flows and high surface tension. Approximated solutions recovered by injecting Gaussian perturbations overgrow, in addition to triggering concentric breathing features that split the medium into high and low-density domains. Secant perturbations on the other hand scatter slowly and form patterns of non-uniformly distributed peaks for strong flows and high surface tension. These results emphasize that Gaussian perturbations strongly modulate the activity of bacteria, hence can be exploited to perform fast spreading in environments with changing properties. In this sense, Gaussian profiles are better candidates to explain quick bacterial responses to external factors. Secant-type approximated solutions slowly modulate the bacterial activity, hence are better alternatives to dive into weak bacterial progressions in heterogeneous media.

## Introduction

Chemotaxis, the directed motion of microorganisms toward/away attractive/repulsive chemical compounds is highly encountered in the biological realm. It has been studied in homogeneous settings, yet in reality, the biological environments within which cells are immersed are very complex due to their composition. This says biological media embed several components that significantly impact the functions and activities of living biological entities in those media. However, the wide range of functions induced by the medium composition on chemotactic particles remains poorly understood. The latter observation urges the need to reassess chemotaxis in media exhibiting heterogeneous properties.

In their natural habitat, the external forces exerted upon chemotactic microorganisms monitor their collective transport and regulate the geometric patterns they form^1^. At the individual level, the run-and-tumble motion exhibited by bacteria leads to thinking that those external forces push bacteria out of equilibrium. In active matter systems, such a mechanism has been shown to generate large density fluctuations^2,3^, and can also trigger several types of instabilities^4,5^. Once they are initially inoculated at a location in the petri dish device, bacteria aggregate into patterns of different sizes and textures, hence creating domains of high and small densities. Such separation into rich and poor density domains is now known to happen when the bacterial spreading speed decreases rapidly with the density^6,7^. It is therefore reasonable to think that patterns emerging from bacterial assemblies are somehow steady^7^, hence the crucial interest in identifying key processes involved in their development.

In non-homogeneous media such as gels, tissues, soils, and sediments, for instance, cells squeeze through lacunae and thereby spread through the whole body^8–11^. Exploiting the non-homogeneity property of the medium also provides an alternative route to hold back the progression of metastatic cancerous cells^12,13^. In agriculture, bacteria responsible for the protection of plant roots move through soils, hence concomitantly increasing crop productivity of farmers^14,15^. Similarly, in the efforts to improve bioremediation, it has been shown that motile bacteria can be used to degrade contaminants trapped in groundwater^16,17^. All the above-cited examples describe configurations where movements of active biological particles in complex and porous media may be used to optimize their potential use. In this sense, the observation of a wide range of behaviors of bacteria in those media has instigated several phenomenological models^7,18–25^. In those models, the non-homogeneous aspect of the medium was modeled through a nonlinear or density-dependent diffusivity of active entities. By considering a linearly density-dependent diffusion, the authors in^18^ introduced a modified Keller-Segel model for chemotaxis. In that model, bacterial aggregation happens when the competition strength between diffusion and chemical interaction is above a critical threshold, in addition to the fact that high-low density transition remains continuous. However, real experimental situations shed the light on a more complicated density-dependent dispersal^26,27^. Under the latter configurations, the resulting chemotaxis models have been shown to admit weak and global solutions^19,20,22,23^, yet they did not provide explicit analytical formulas or those solutions. In the case of a density-dependent diffusion obeying a power law nonlinear evolution, a stable speed propagation was found in addition to the fact that the compact support through which propagation happens may shrink when chemoattractant concentration exhibits a specific profile^20^. However, considering a diffusion rate whose variations follow a power nonlinearity implies that cell diffusion goes to infinity at high densities. In real experiments, patterns are formed at high bacterial aggregation and hence at a minimal spreading speed^1,6,7,28^. It is therefore reasonable to think that a density-dependent diffusion following power law evolution can be physically problematic because it may lead to an infinite spreading speed or a fast spreading at higher density. An increasing diffusion rate is very different from classical diffusion in the sense it may not imply the active movement of diffusing species from high to low densities. Experimental observations in^26^ reveal that when inoculated at an initial point, cells move faster. As they spread towards outer boundaries, their spreading speed decreases as well as their density, implying that bacterial diffusion decreases with the density. This is well in line with results obtained experimentally by considering a constant bacterial diffusion^1,29–31^. Therefore, a decreasing density-dependent diffusion is a better choice to describe the biological realities of pattern formation. Those effects have been modeled as volume filling process^25^. The authors used their model to prove a steady propagation of bacteria at a finite speed.

Experimental observations of E. coli cells in porous media reveal that they move in an intermittent and transient fashion way in space with traps^10,32^. If a cell is trapped, then its response to an external stimulant depends on its trapping site, hence may be delayed. The latter effect can be accounted for in a model by introducing a long-range diffusion^1,28,33^. Also, in their natural habitat, cells are immersed in fluids. Although the fluid constitutes the compact support upon which propagation of traveling wave structures is made possible^28,34^, the above mentioned-models did investigate its contribution to the evolution of chemotactic particles. In active suspensions, experimental evidence suggests that hydrodynamic interactions guide traveling bands of bacteria at finite speed^2–4,35^, emphasizing the role played by fluid flows on the transport of cells. Some of these studies were carried out either in a uniform fluid environment or in an incompressible Navier fluid. However, the spatial reorganization of constituents of an experimental setting is largely shaped by fluid flows which have the capacity to enhance heterogeneity. More importantly, turbulent flows are known to stretch nutrients patches hence creating strong gradients at the microscopic level^36^. Fluid flows are highly relevant in the engineering of a broad range of natural processes including biochemical cycling to remineralization and bioremediation^16,17^, yet how chemotactic bacteria are transported in porous media exhibiting a complicated fluid flow remains poorly understood. In this study, we are concerned with a fluid whose strength varies with space, and that can be easily realized in real experiments. Investigations of chemotaxis models under the latter configuration remain a research domain of interest. Inspired by the limitations cited above we introduce a mathematical model for chemotaxis in two-dimensional settings. The model we introduce describes the dynamics of chemotactic particles in complex and the dispersal of those particles does not obey a power law nonlinearity. Such an inhomogeneous system has not yet received any theoretical analysis, to the best of our knowledge. Through a modified linear stability analysis, we dig into the spatiotemporal evolution of localized structures.

The remainder of our discussion proceeds as follows. In “Results” section, we present the model for the chemotaxis phenomenon and discuss its inherent biological realities. In “Numerical results” section, the linear stability analysis of our model is performed by the means of generalized localized perturbations. Later on, approximated solutions of the proposed model are constructed. We numerically test the sensitivity of our system under several parametric conditions and provide the biological implications of our observations. “Conclusion” section summarizes the manuscript.

## Results

### Modeling chemotaxis in non-homogeneous media

In our description, we are concerned with cells whose moving speed depends on the local cellular density, in addition to the advective flows imposed by the medium. In complex fluids, the finiteness of cell volume affects their spreading in addition to triggering a nonlocal response of cells to external stimuli. Although the latter was shown to be responsible for long-range diffusive effects^1,28^, it has also been linked to the generation of surface tension in a bacterial community^7^. The resulting mathematical model reads^1,7,28–31^.

1a $$\begin{aligned} \frac{\partial n }{\partial t} + \mathbf {\nabla } \cdot \left( {\textbf{u}}({\textbf{r}})\cdot n\right)&= \mathbf {\nabla } \cdot \left( D(n)\mathbf {\nabla }n\right) - \mathbf {\nabla } \cdot \left( \chi (n,c) \mathbf {\nabla } c\right) - d_2 \nabla ^4 n + k_3n\left( \frac{k_4s_0^2}{k_9 + s_0^2} - n\right) , \end{aligned}$$

1b $$\begin{aligned} \frac{\partial c }{\partial t} + \mathbf {\nabla } \cdot \left( {\textbf{u}}({\textbf{r}})\cdot c \right)&= D_c \nabla ^2 c + \frac{k_5s_0n^2}{k_6 + n^2} - k_7 nc. \end{aligned}$$

In the model Eq. (1), n and c are respectively bacterial density and chemoattractant concentration. c and n severally diffuse at $D_c$ and $D(n) = \frac{v_0^2\tau }{d}(1 - \frac{\lambda n}{2})e^{-\lambda n}$ rates. $v_0$ is the initial velocity of a given bacterial population whose average tumbling time is $\tau$. $\lambda$ is a constant parameter that ensures speed reduction due to the crowding of cells. d represents the spatial dimension, d = 1 in one dimension, d = 2 in two dimensions, and d = 3 in three dimensions. $\chi (n,c) = \frac{k_1 n}{(k_2 + c)^2}$ measures the bacterial sensitivity to the chemical c. $k_1$ quantifies the strength of the entailed chemotactic motion while $k_2$ accounts for the fact that the chemotactic motion saturates in high-concentration fields. ${\textbf{u}}({\textbf{r}}) = (u,v)^T$ describes a two-dimensional spatiotemporal complex fluid within which cells are immersed. The present study considers the case that the velocity field ${\textbf{u}}({\textbf{r}})$ is known and well under control as in the experiments reported in^35,37–39^. This means that the experimenter has the freedom to monitor the strength as well as the nature of the flows. The above assumption suggests the choice of an analytical expression of the fluid velocity field ${\textbf{u}}({\textbf{r}})$ that mimics with quite a good accuracy experimental observations, although it may be interesting to include momentum and state equations in the model Eq. (1). The latter fact does not fall in the scope of this work and will be carried out elsewhere. ${\textbf{r}} = (x,y)^T$ the two-dimensional position vector. The velocity field ${\textbf{u}}({\textbf{r}})$ will be chosen such that its magnitude matches theoretical^1,28^ as well as experimental^35,37–39^ observations. During their motion, bacteria exert mechanical forces upon their surrounding. The intensity of those forces varies with the position of an arbitrary individual. Thus, the bacterium-bacterium interactions contribute to the global response and subsequently the mechanisms through which an individual probes the environment. The contribution of bacteria-bacteria interactions to the bulk bacterial response can be mathematically understood as a non-local action. For bacterial distribution exhibiting symmetric kernels, it has been demonstrated that the latter non-local action is equivalent to variations of gradient response of bacteria over a unit surface^1^. In other words, particle-rich and particle-depleted domains exert compressive actions upon each other as described by the continuum theory of multi-phase flows^40^. In the model Eq. (1) those effects are measured by the parameter $d_2$. In Eq. (1a), $k_3$ is the proliferation rate of cells, and $s_0$ is the substrate concentration. Note that in this description of chemotaxis, substrate abundance is a key factor in determining the maximum carrying capacity of the medium. $k_4$ is the rate at which cells consume substrate and $k_9$ is the saturation parameter ensuring that cell density remains below the carrying capacity. The two last terms of Eq. (1b) severally account for chemoattractant production and consumption. Hereafter, for sake of simplicity, we set $f(n,c) = k_3n\left( \frac{k_4s_0^2}{k_9 + s_0^2} - n\right)$ and $g(n,c) = \frac{k_5s_0n^2}{k_6 + n^2} - k_7 nc$^1^. Except for the surface tension $d_2$, the velocity field ${\textbf{u}}$, the volume filling $\lambda$, and the kinetic parameters $k_5, k_6, k_7$ that are unknown, numerical values of all the other parameters of the Eq. (1) are tabulated in^1^ and will be accordingly used here without further modifications. We provide in Table 1 those values.

**Table 1:** Parameter network values.

Parameters	Values and units	References
$D_n = v_0^2\tau$	$2-4\cdot 10^{-6}$ ${\text{cm}}^2 \, {\text{s}}^{-1}$	^30^
$D_c$	$8.9\cdot 10^{-6}$ ${\text{cm}}^2 \, {\text{s}}^{-1}$	^30^
$k_1$	$3.9\cdot 10^{-9}$ ${\text{M}} \, {\text{cm}}^2 \, {\text{s}}^{-1}$	^41^
$k_2$	$5\cdot 10^{-6}$ *M*	^41^
$k_3$	$1.69\cdot 10^{-9}$ ${\text{hr}} \, {\text{ml}}^{-1} \, {\text{cell}}^{-1}$	^31^
$k_4$	$3.8\cdot 10^{8}$ cells ${\text{ml}}^{-1}$	^31^
$k_9$	$4\cdot 10^{-6}$ ${\text{M}}^2$	^31^
$s_0$	$1-3\cdot 10^{-3}$ M	^31^

Further, we introduce the dimensionless parameters

2 $$\begin{aligned} n^*&= \frac{n}{\sigma }, \quad c^* = \frac{c}{c_0}, \quad t^* = \frac{k_5s_0}{k_2}t, \quad \mathbf {\nabla }^* = \left( \frac{k_5s_0}{D_ck_2}\right) ^{\frac{1}{2}}\cdot \mathbf {\nabla }, \quad {\textbf{u}}^* = \left( \frac{k_2}{D_ck_5s_0}\right) ^{\frac{1}{2}}\cdot {\textbf{u}}, \quad \alpha = \frac{v_0^2\tau }{dD_c}, \quad \Phi = \frac{\lambda \sigma }{2}, \nonumber \\ \chi _0 &= \frac{k_1}{D_ck_2}, \quad D_2 = \frac{d_2k_5s_0}{k_2D_c^2}, \quad r = \frac{k_2k_3\sigma }{k_5s_0}, \quad \mu = \frac{k_6}{\sigma ^2}, \quad \beta = \frac{k_2k_7\sigma }{k_5s_0}, \quad \sigma = \frac{k_4s_0^2}{k_9 + s_0^2}. \end{aligned}$$

Omitting the asterisks for sake of simplicity, transforms the model Eq. (1) into

3a $$\begin{aligned} \frac{\partial n }{\partial t} + \mathbf {\nabla } \cdot \left( {\textbf{u}}({\textbf{r}})\cdot n\right)&= \alpha \mathbf {\nabla } \cdot \left[ \left( 1 - \Phi n\right) e^{-2\Phi n} \mathbf {\nabla }n\right] - \chi _0 \mathbf {\nabla } \cdot \left[ \frac{n}{(1 + c)^2}\mathbf {\nabla } c\right] - D_2 \nabla ^4 n + rn\left( 1 - n\right) , \end{aligned}$$

3b $$\begin{aligned} \frac{\partial c }{\partial t} + \mathbf {\nabla } \cdot \left( {\textbf{u}}({\textbf{r}})\cdot c \right)&= \nabla ^2 c + \frac{n^2}{\mu + n^2} - \beta nc. \end{aligned}$$

Experiments on chemotaxis are generally performed in a confined domain that we denote by $\Omega$ in the present investigations. Further, zero flux can be experimentally realized at the boundaries $\partial \Omega$ is considered. Experiments generally start with a bacterial density and chemoattractant concentration that are both non-null at the initial time. If the outward normal derivative at the boundaries $\partial \Omega$ if $\frac{\partial }{\partial \nu }$, then above-mentioned conditions are mathematically translated by

$$\begin{aligned} n({\textbf{r}},t = 0) = n_0({\textbf{r}}), \quad c({\textbf{r}},t = 0) = c_0({\textbf{r}}), \quad \forall x,y \in \Omega . \quad \text {And,}\quad \frac{\partial n}{\partial \nu } = \frac{\partial c}{\partial \nu } = 0 \quad \forall x,y, \in \partial \Omega \nonumber \end{aligned}$$

The former’s denote the initial condition associated with Eq. (3) while the latter indicates its boundary conditions.

Except for the velocity field ${\textbf{u}}({\textbf{r}})$, the surface tension $D_2$, and the overcrowd inhibitor parameter $\Phi$, all parameters in the dimensionless model Eq. (3) are well known and given in^1^. In the latter reference, Murray discussed several chemotaxis models in one and two spatial dimensions. Those models do not take into consideration the fact that cells are generally immersed in fluids, yet it was recently proved that the fluid constitutes the compact support upon which propagation of coherent structures in chemotactic systems occurs^28,34^. In absence of the surface tension ($D_2 = 0$), overcrowding ($\Phi = 0$) and velocity field (${\textbf{u}} = 0$), Eq. (3) reduce to a chemotaxis model with a constant diffusion rate that has been widely discussed^1,29–31^. The surface tension parameter $D_2$ also known to play the role of a long-range diffusive term has been shown to exert stabilizing $D_2 > 0$ or destabilizing $D_2 < 0$ forces on a single reaction-diffusion equation with constant parameters^1^. For $D_2 > 0$, the same observation holds for a set of nonlinearly coupled partial differential equations describing the long-ranged invasion of cancerous cells in biological tissues^33^. Although long-range diffusion enhances the stability of reactive systems, we recently proved that such enhancement is not necessarily accompanied by an increase in the number of cells transported^28^. Those results brought a substantial understanding of the role of surface tension, albeit they were derived in situations where cell diffusion is constant, and the velocity field being uniform. Experimental investigations proved the dependence of diffusion on the density of the spreading species^26,27^. Generally speaking, a positive diffusion rate implies a spreading towards regions of weak distributions. The density-dependent diffusion rate considered here implies the confined spreading at a finite speed, a feature that has been associated with pattern formation and evolution^7^. In Eq. (3), the latter observation is ensured for values of $\Phi$ that are always smaller than a threshold $\Phi _{th} = \frac{1}{n_{th}}$. A density-dependent diffusion attests to the porosity of the medium and may lead to degeneracy^7,19,20^. In their analyses of chemotaxis in porous media, the authors in^37–39,42,43^ combined experimental and mathematical modeling, and proposed several conditions within which an enhanced collective transport of chemotactic bacteria may be observed in two-dimensional settings. The models they introduced consider a constant porosity, yet it is well known that dispersal in a medium intrinsically depends on the density of the spreading species in that medium. Further, prominent works on chemotaxis^1,7,29–31,37–39,42,43^ did not account for long-range diffusion and birth-death processes simultaneously, which implies that they minimized some aspects of bacterial activities. Investigations reported in^37–39,42^ neglected proliferation, an assumption that may be problematic because it is equivalent of assuming that the timescales for experimental observations are short although they worked with flow rates of orders of a few meters per day (very steady flows). This is not always the case for every bacterium. For example, *E. coli* duplication time is 20 minutes in rich aerobic environments. Therefore, carrying out an experiment with E. *coli* with the flow rate used in^37–39,42^ under very short timescales would be equivalent to not taking into account the contribution of the fluid flows on the collective migration of those bacteria. In confined domains, the authors in^44^ showed that proliferation is the leading force that drives spreading when confinement is increased. The latter results were derived in settings where fluid flows, surface tension, and a density-dependent diffusion rate were not taken into consideration, yet their conclusion urges to need to account for those aspects in order to advance our understanding of collective bacterial spreading in heterogeneous media. In this regards, those models present some gaps that are well-filled by Eq. (3). Recently, chemotaxis models with density-dependent power-law nonlinearity were introduced^19,20^ to determine the global conditions within which chemotaxis models are stable. Yet if not well-scaled, using a power-law nonlinear diffusion may lead to nonphysically relevant features of unbounded growth and spreading at infinite speeds. Experimental as well as theoretical results suggest that chemotaxis can yield phase-separated states with decreasing speeds^1,7,29–31,45^. In absence of chemotaxis, proliferation, and surface tension, the density-dependent diffusion rate considered in this paper ensures a decreasing velocity field, hence is physically relevant to probe the collective dispersal of chemotactic entities in porous media. The simultaneous presence of the surface tension, proliferation, density-dependent diffusion, and nonuniform flow in Eq. (3) is suspected to be the seat of interesting spatiotemporal behaviors that, to the best of our knowledge remain to be investigated. To our knowledge, theoretical and experimental models incorporating these parameters have not yet been proposed. In this sense, Eq. (3) constitute a generic model that can be used to improve our understanding of collective chemotactic transport in porous media.

### Stability of localized perturbations

We perform the stability analysis of Eq. (3) by considering small deviations about the homogeneous steady state $n({\textbf{r}},t) = n_0 + \varepsilon N_1 e^{p({\textbf{r}},t)} + c.c, c({\textbf{r}},t) = c_0 + \varepsilon C_1 e^{p({\textbf{r}},t)} + c.c.$ $\varepsilon$ is the perturbation parameter, *c*.*c* represents the complex conjugate of the preceding terms, and $p({\textbf{r}},t)$ is a generalized Spatio-temporal complex function that can be written in the form $p({\textbf{r}},t) = p_r({\textbf{r}},t) + i\cdot p_i({\textbf{r}},t)$. Inserting the above ansatz into Eq. (3) and keeping only linear terms in $\varepsilon$ leads to a quadratic equation, whose solutions for the variable $\frac{\partial p}{\partial t}$ reads

4 $$\begin{aligned} \frac{\partial p }{\partial t}&= \frac{1}{2} \left[ -\Re (a_{11} + a_{22}) \pm \sqrt{\frac{\Delta _r + |\Delta |}{2}} \right] + \frac{i}{2} \left[ -\Im (a_{11} + a_{22}) \pm \sqrt{\frac{|\Delta | - \Delta _r }{2}} \right] - {\textbf{u}}({\textbf{r}})\cdot \mathbf {\nabla }p - \mathbf {\nabla }\cdot {\textbf{u}}({\textbf{r}}) \end{aligned}$$

The parameters $a_{11}, a_{22},$ and $\Delta = \Delta _r + i\Delta _i$ appearing in Eq. (4) are defined in Appendix A of the supplementary file. Equation (4) are significantly important since they indicate that the nature of the medium within which cells are immersed directly influences the growth rate of the perturbations. In an incompressible fluid, for instance, the last term of Eq. (4) cancels. Thus the experimenter can suppress fluid effects by applying transversal perturbations ${\textbf{u}}({\textbf{r}})\cdot \mathbf {\nabla }p = 0$. In other words, the perturbation must not be parallel to the fluid velocity field if one intends to perceive significant effects of incompressible fluids on the stability of system Eq. (3). However, Eq. (4) indicate that the latter condition is not sufficient to inhibit the effects of compressible flows on the stability of the system. In order to see how far that goes, we consider only temporal perturbations. Following the fact that the system is stable when $\Re \left( \frac{\partial p}{\partial t}\right) < 0$, one arrives at the stability criteria

5 $$\begin{aligned} F_n + G_c - 2\cdot \mathbf {\nabla }\cdot {\textbf{u}}({\textbf{r}})< 0, \quad G_n\cdot F_c - F_n\cdot G_c\ + \left( F_n + G_c\right) \cdot \mathbf {\nabla }\cdot {\textbf{u}}({\textbf{r}}) < 0. \end{aligned}$$

In incompressible media, Eq. (5) reduces to the Turing stability criterion which has largely been discussed by Murray in his seminal book^1^. Hence Eq. (5) represent the Turing stability criterion for chemotactic particles in compressible fluids when submitted to temporal perturbations. It says that the instability of the system is not only the byproduct of a coupled dynamic between the cell’s activity and chemical reactions. When submitted to Spatio-temporal perturbations, the system is stable if

6 $$\begin{aligned} G_nF_c - \Re (a_{11})\Re (a_{22}) - \chi _0 G_n \Re (a_{12}) - 2\Re (a_{11} + a_{22})\left[ \mathbf {\nabla }\cdot {\textbf{u}}({\textbf{r}}) + {\textbf{u}}({\textbf{r}})\cdot \mathbf {\nabla }p_r({\textbf{r}},t) \right] + \frac{\Im (a_{11})\Im (a_{22}) }{2} < 0. \end{aligned}$$

Equation (6) generalizes the set of inequalities Eq. (5), and gives a stability criterion of the model Eq. (3) when under the influence of slowly varying generalized perturbations. Eq. (6) does not depend on the specific form of the perturbation, hence it may be considered as a generalized stability criterion for chemotactic particles in complex fluids. It indicates that the localization of stability-instability transition points is a complex interplay between kinetics undergone by cells, the type of perturbations, and the nature of the flow within which cells are immersed. In other words, the nature of the medium plays an intrinsic role in deciphering where the system is globally stable. This calls for the experimenter to be careful about the choice of the fluid, in order for him to predict with quite a good accuracy how and where chemotactic cells will exhibit a collectively stable dynamic. For sake of simplicity, we consider a velocity field $${\textbf{u}}({\textbf{r}}) = {\textbf{u}}(x,y) = \frac{x}{\tau _x}\mathbf {e_x} - \frac{y}{\tau _y}\mathbf {e_y}$$, where $$\mathbf {e_x}$$ and $$\mathbf {e_y}$$ are the unit vectors along x and y directions, respectively. $$\tau _x$$ and $$\tau _y$$ are parameters whose values can be used to separate compressible ($$\tau _x \ne \tau _y$$) and incompressible flows ($$\tau _x = \tau _y$$). The latter constitutes the configuration within which our results are discussed below. Such a velocity field may be used to describe flows of elliptic/circular gas of uniformly distributed fluid particles. In reality, coupling fluid and chemotactic bacteria requires the introduction of fluid equations in the model Eq. (3). Here we suppose the external fluid to be known, hence the exact determination of the fluid velocity field is beyond the scope of this work. Experimental investigations on the chemotactic behaviors of Dictyostelium discoideum in fluids reveal that the strength of the flows within which cells are immersed has deep effects on the speed and wave structures ensuring the transport of those cells^35^. Similar conclusions were reported in the studies of the collective migration of bacteria in a stratified heterogeneous medium^37–39^. Falling on the line with these observations, the velocity field considered here exhibits strong ($$\tau _x, \tau _y < 1$$) and weak ($$\tau _x, \tau _y > 1$$) flows, corresponding to high and small values of $$|{\textbf{u}}(x,y)|$$, respectively. Furthermore, we draw from Eq. (6) that stability-instability transition points non-trivially depend on the space and time variables. Investigating instability features under those configurations becomes computationally demanding. An issue that can be circumvented by assuming perturbed solutions around the steady states at the initial time $$n({\textbf{r}},0) = n_0 + \varepsilon N_1 e^{p({\textbf{r}},0)} + c.c = n_0 + \varepsilon \cdot \cos \left[ p_i({\textbf{r}},0)\right] \cdot \exp \left[ p_r({\textbf{r}},0)\right] , c({\textbf{r}},0) = c_0 + \varepsilon C_1 e^{p({\textbf{r}},0)} + c.c = c_0 + \varepsilon \cdot \cos \left[ p_i({\textbf{r}},0)\right] \cdot \exp \left[ p_r({\textbf{r}},0)\right]$$. With the above-considered ansatz, the complete determination of the instability growth rate $$\left[ p_{r_t}(x,y,0) = \Re \left( \frac{\partial p}{\partial t}|_{t = 0}\right) \right]$$ using Eq. (4) requires to know the expressions of $$|\mathbf {\nabla }p({\textbf{r}},0)|, 
\mathbf {\nabla }^3p({\textbf{r}},0), \nabla ^2p({\textbf{r}},0), \nabla ^4p({\textbf{r}},0)$$. The power of the method comes from the fact it supposes $$p({\textbf{r}},t)$$ to be known at the initial time t = 0. Following the analyses performed in^46,47^ we initially choose $$p({\textbf{r}},t = 0)$$ such that the amplitude of the perturbation is a spatially localized distribution. In this study, we set $$p({\textbf{r}},0) = i\cdot {\textbf{k}}\cdot {\textbf{r}} + \log \left[ L({\textbf{r}})\right] .$$ $${\textbf{k}} = (k_x,k_y)$$ being a two-dimensional perturbation wave vector. Using the latter expression, the complete characterization of the system’s stability requires analyzing the growth rate, phase, and envelope speeds of the injected perturbations. In the two-dimensional framework considered here, those parameters are severally given by^46–48^

7 $$\begin{aligned} p_{r_t}(x,y,0)&= \Re \left[ \frac{\partial p(x,y,t)}{\partial t}|_{t = 0}\right] , \quad V_{ph_x}(x,y,0) = \frac{1}{k_x} \Im \left[ \frac{\partial p(x,y,t)}{\partial t}|_{t = 0}\right] , \quad V_{ph_y}(x,y,0) = \frac{1}{k_y} \Im \left[ \frac{\partial p(x,y,t)}{\partial t}|_{t = 0}\right] , \nonumber \\ V_{en_x}(x,y,0)&= \frac{\Re \left[ \frac{\partial p(x,y,t)}{\partial t}|_{t = 0}\right] }{\frac{\partial \Re [p(x,y,0)]}{\partial x}}, \quad V_{en_y}(x,y,0) = \frac{\Re \left[ \frac{\partial p(x,y,t)}{\partial t}|_{t = 0}\right] }{\frac{\partial \Re [p(x,y,0)]}{\partial y}} \end{aligned}$$

To be precise, $V_{ph_x}(x,y,0)$ and $V_{ph_y}(x,y,0)$ are the phase speeds along x and y axes, respectively. Seemingly, $V_{en_x}(x,y,0)$ and $V_{en_y}(x,y,0)$ severally represent the envelope speeds of perturbations along the x and y-axis. We analyze these parameters by considering two different localized distributions $L({\textbf{r}}) = \left\{ e^{-\left( \frac{x - x_0}{\sigma _x}\right) ^2}\cdot e^{-\left( \frac{y - y_0}{\sigma _y}\right) ^2}, \cosh ^{-1}\left( \frac{x - x_0}{\sigma _x}\right) \cdot \cosh ^{-1}\left( \frac{y - y_0}{\sigma _y}\right) \right\}$. $(x_0,y_0)$ represents spatial coordinates of the centre of those distributions and, $\sigma _x, \sigma _y$ measure the widths of the distributions along the x and y directions, respectively.

**Figure 1:**
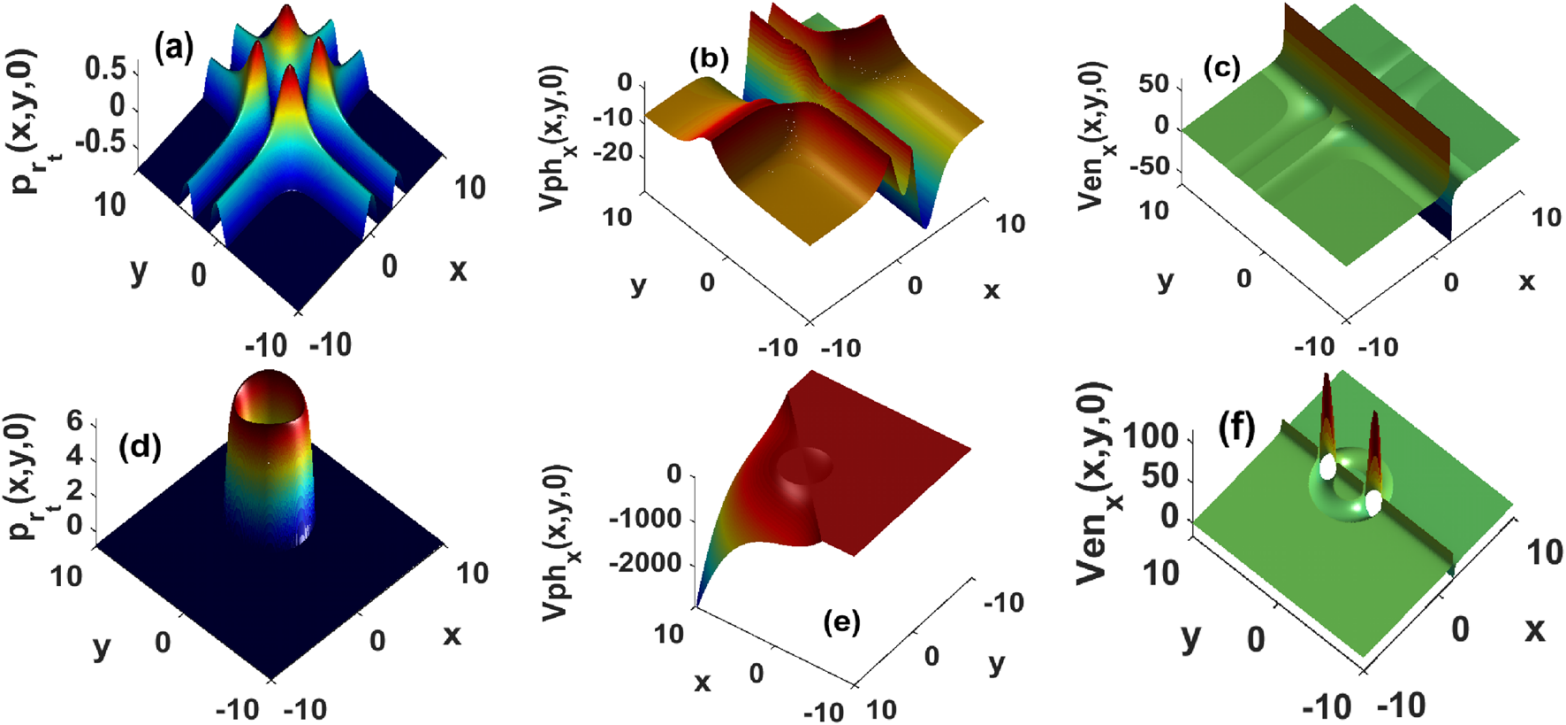
Growth rate, phase and envelope speeds of the localized perturbations in absence of a background velocity field ($u = v = 0$) $D_2 = 1.62, \sigma _x = \sigma _y = 2, k_x = k_y = 0.05, x_0 = y_0 = 2, \Phi = 2\cdot 10^{-3}$. Top panels are obtained with the secant function, and bottom panels are related to the Gaussian distribution.

The envelope speeds along the x and y-spatial directions, corresponding to the two last expressions in Eq. (7) reveal the existence of a singularity at the centre of the distribution. The singularity draws a net separation between regions where the envelope speeds are either positive or negative. This is consistent with our previous observations in a two-population chemotaxis model, where singularity appears as a key element in distinguishing domains where expansive ($V_{en} < 0$) or compressive ($V_{en} > 0$) envelopes are generated^47^. Figure 1c,f depict the envelop speed corresponding to secant and Gaussian perturbations, respectively. It is observed that Gaussian profiles propagate faster than their secant counterpart. Moreover, in the region x < 0, envelope speeds increase with the distance and reach infinity near the singularity line Fig. 1c,f. This means a fast propagation at the centre of the distribution is expected. In other words, a group of cells travels faster at locations where the injected perturbations are maximal, an observation that is consistent with experimental conclusions^26^. Around the singularity, the absolute value of the envelope speed decreases with the distance, leading to think that cells continuously deposit their energy in the medium through frictions and interactions. We propose such an energy deposition in an active system to be the signature of a phase separation mechanism. Those results indicate that starting from the centre of each distribution, compressive (expansive) envelopes propagate towards left (right) boundaries with decreasing speeds. As the wave approaches the boundaries, the envelope speed goes to zero, implying either a zero or a very steady propagation in the far field. Under the same parametric conditions, panels Fig. 1b,e show the phase speed of the distribution remains continuous and is negative over the spatial domain under consideration. For the Gaussian profile Fig. 1e, the absolute value of the phase speed increases and reaches its peak at the edge of boundaries. The implications are that, as the band of cells spreads across the medium, the intensity of its vibrations increases towards the boundaries. Meanwhile, the hyperbolic secant function indicates that in addition to being finite, those high vibrations are sparsely distributed across the medium Fig. 1b. The instability growth rate corresponding to secant and Gaussian profiles are depicted in panels Fig. 1a,d, respectively. The localized perturbations trigger the emergence of unstable structures originating from the centre of those distributions. In other words, when the system is more likely to be unstable at locations where perturbations reach their peaks. However, the secant perturbation reveals that stable patterns are expected to emerge at long distances Fig. 1a. Panel Fig. 1d draws quite a different feature, emphasizing that Gaussian perturbations are more likely to push the steady out of equilibrium across the whole spatial domain.

**Figure 2:**
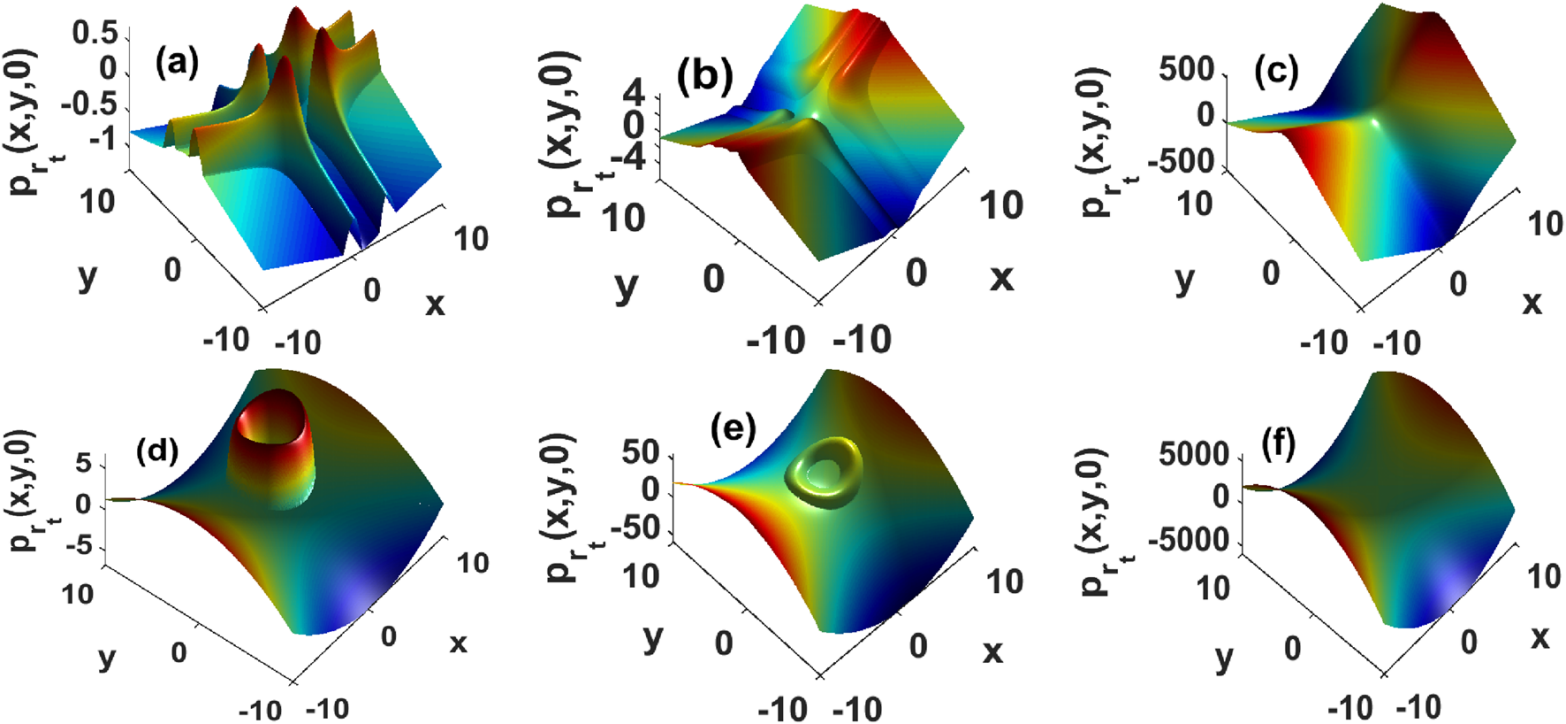
Reduction of instability domains in presence of a strong incompressible velocity field. Top panels are obtained by injecting two-dimensional hyperbolic secant distribution, and bottom panels are recovered by using a two-dimensional Gaussian profile. Left panels $\tau _x = \tau _y = 10^2$, middle $\tau _x = \tau _y = 1$ and right panels $\tau _x = \tau _y = 0.01$. Other parameters as in Fig. 1.

**Figure 3:**
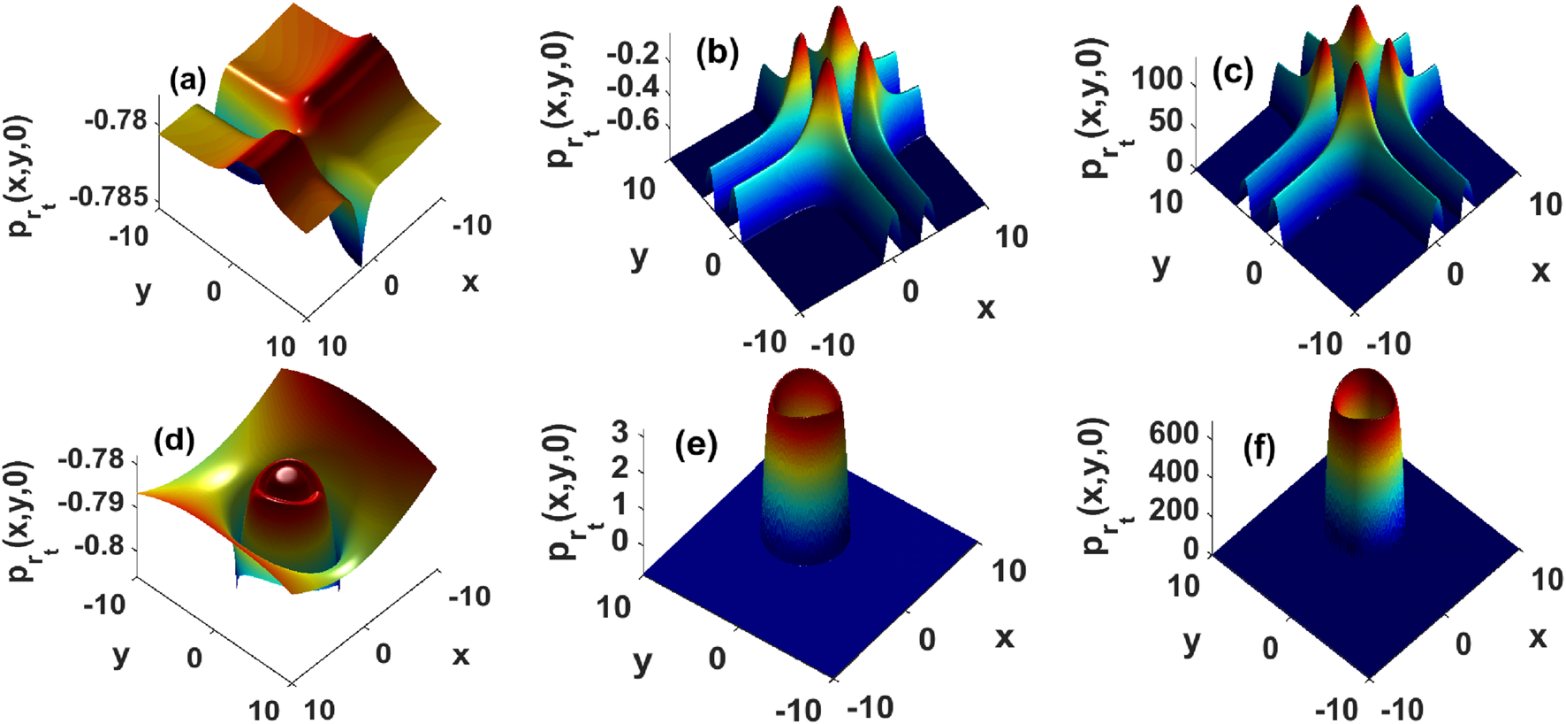
Spreading of unstable domains with surface tension, and for weak incompressible flows $\tau _x = \tau _y = 10^2$. Top panels are obtained by injecting the hyperbolic secant distribution, and bottom panels are derived using a Gaussian profile. Left panels $D_2 = 1.62\cdot 10^{-3}$, middle $D_2 = 1.62$ and right panels $D_2 = 162$. Other parameters as in Fig. 1.

Results displayed in Fig. 1 have been derived in absence of fluid flows ${\textbf{u}}({\textbf{r}}) = {\textbf{0}}$. In presence of advection, Figs. 2 and 3 show that the growth rate of instability is significantly modified. For instance, even weak flows shift the stability domains as well as the magnitude of the perturbation growth rate Fig. 2a,d. In Fig. 2, the strength of the flows increases from left to right panels. Progressively, the instability peaks located at the center of the distribution are progressively wiped-out. The magnitude of instability growth rate increases with flows and unstable patterns are expected to rise along the y-axis, while stable structures are predicted to be generated along the x-axis. Consequently, fluid flows favor the emergence of heterogeneous structures that do not solely depend on the strength of the chemical gradient in the medium or the surface tension cells exert upon their neighboring, hence corroborating results presented in^10^. The high magnitudes of the growth rate in presence of strong flows imply a fast decay/growth of perturbations in the stable/unstable regions. In the stability regions, strong stationary patterns form, providing the advantage to improve bacterial response to physiological mechanisms such as drug delivery. The instability triggers large fluctuations about the steady state, hence a density increase that is reminiscent of features reported in Belousov-Zhabotinsky reactions^49^. We can thus argue that unstable regions rise because strong flows quickly advect the components of the medium that are responsible for creating the conditions within which the bacterial density converges towards a stationary state. These results complement experimental and theoretical investigations in^35^, where the authors discussed how flows can be chosen to monitor the collective transport of chemotactic bacteria in fluids, although their analysis does not take into consideration the density dependence of cellular diffusivity and surface tension. In weak flows, Fig. 3 reveals how surface tension affects growth diagrams. Interestingly, unstable regions rise and span the domain with the strength of the surface tension Fig. 3. Although surface tension may exert stabilizing forces in reactive systems^1,7,33^, panels of Fig. 3 confirm the existence of an upper threshold above which the surface tension has destabilizing effects. Physiologically meaning, interactions between particle-rich and particle-depleted areas are important to control stability, but the strength of those interactions must remain small enough so that the resulting collective transport operates through stable structures.

## Numerical results

### Weakly varying solutions

The stability analysis of localized perturbations discussed above suggests that instability may manifest itself either as a fast-spreading or an unbound growth of the bacterial density and chemoattractant concentration wave amplitudes. To test such a hypothesis, we assume weakly varying solutions of Eq. (3)

8a $$\begin{aligned} n({\textbf{r}},t)&= n_0 + \varepsilon \cdot L\left( {\textbf{r}} - V_{en}\cdot t\right) \cdot \cos \left( {\textbf{k}}\cdot {\textbf{r}} - V_{ph}\cdot t\right) , \end{aligned}$$

8b $$\begin{aligned} c({\textbf{r}},t)&= c_0 + \varepsilon \cdot L\left( {\textbf{r}} - V_{en}\cdot t\right) \cdot \cos \left( {\textbf{k}}\cdot {\textbf{r}} - V_{ph}\cdot t\right) . \end{aligned}$$

**Figure 4:**
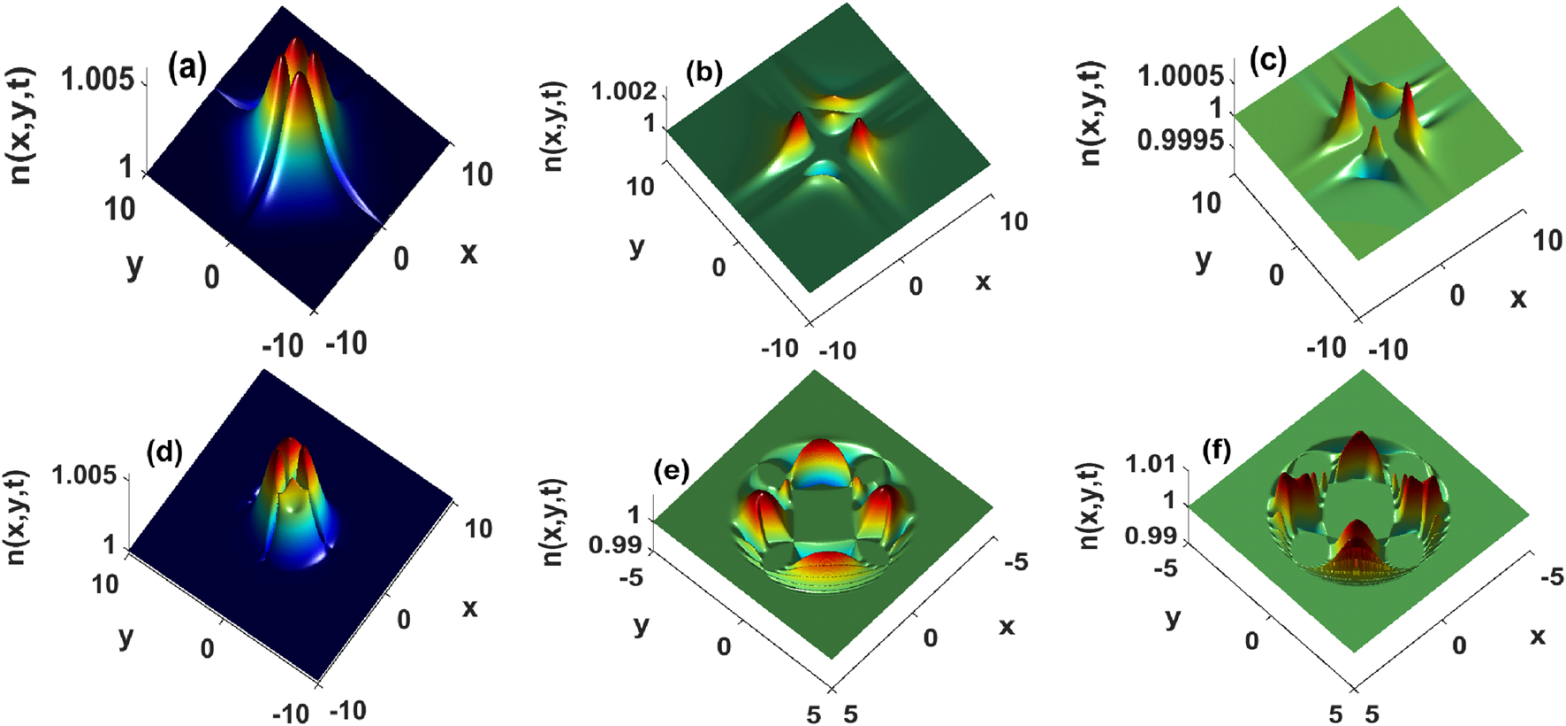
Propagation of bacterial waves at different times. The top panels depict approximate solutions calculated by injecting the hyperbolic secant distribution, and the bottom panels represent solutions derived using a Gaussian profile. $k_x = k_y = 0.05, \tau _x = \tau _y = 10^2, D_2 = 1.62$. Left panels $t = 5\cdot 10^{-3}$, middle $t = 0.5$ and right panels $t = 1$. Other parameters as in Fig. 1.

Equation (8) are used to analyze spatiotemporal evolutions of bacterial densities corresponding to secant and Gaussian perturbations. These functions have been chosen to mimic the pulse-like behavior that was used in experiments reported in^37,38^, and obtained analytically in^28,34,43^. The results displayed in Fig. 4 are indicative of how bacterial wave profiles are modified during propagation as time evolves. The top panels (a)–(c) of Fig. 4 are obtained by injecting secant perturbations. As time goes on, bacterial wave magnitude decreases and converges towards the steady state of the system. In this sense, such a solution is a physically stable object that can be observed in real experiments. Such a profile, therefore, possesses great potential to improve physiological conditions such as fertilization, drug delivery, and organ repairs, just to name a few. In addition, patterns that are initially maximally distributed scatter across the medium, and start breathing weakly (Fig. 4b,c). The scattering leads to a non-uniform redistribution of bacteria at several locations. The non-equal redistribution explains why patterns are not always intrinsically similar, although they may be generated through the same biochemical or interaction mechanisms. The behavior is even more dramatic when Gaussian perturbations are injected in Eq. (8), as depicted in the bottom panels (d)–(f) of Fig. 4. As time increases, the corresponding bacterial wave amplitudes increase, a situation that for a long time leads to unbounded growth or unstable structures. In other words, Gaussian perturbations are more prone to instability, hence confirming the predictions of our stability analysis. Contrary to secant perturbations, Gaussian solutions 4(e)-(f) exhibit strong breathing features during propagation. Those breathers split the medium into regions of high and small bacterial densities, hence giving the opportunity to separate the system into several domains within which cell activity is either high or low. We hypothesize the transition between these two states to set up an intermittent and unsteady motion of bacteria that can be characterized as bacterial turbulence. Interestingly, the spreading toward the boundaries happens in a concentric manner. Cyclic waves exist in chemotaxis^1,29,31^ as well as in reactive systems^7,45^. The concentric wave displayed in Fig. 4e,f are very particular because the bacterial distribution within the circle returns to a uniform steady state. In binary fluids with continuously ramped supersaturation, such regression has been shown to be the onset of spinodal instability^50^. The analysis of the latter dynamical behavior in Eq. (3) is beyond the scope of this work, and will be carried out somewhere else.

**Figure 5:**
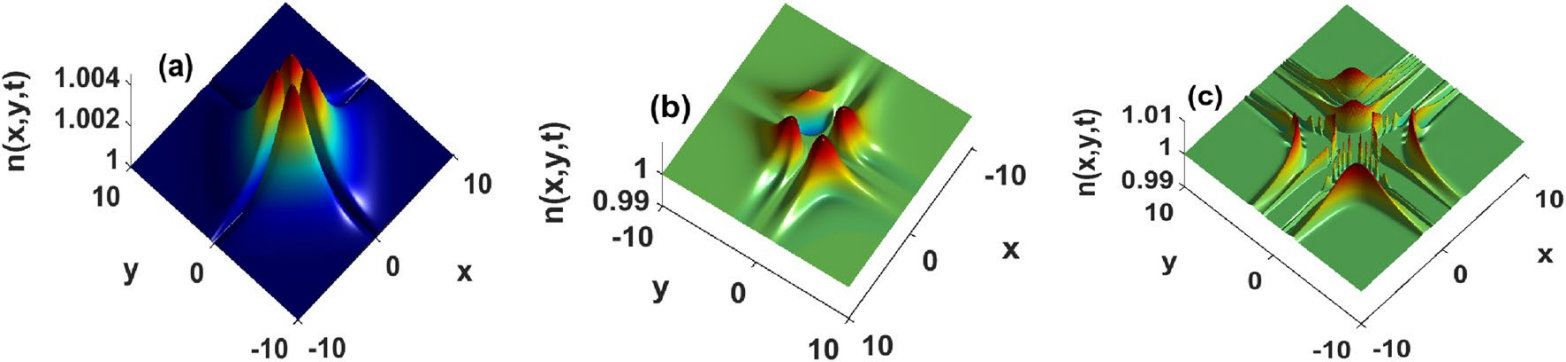
The amplitude of approximated bacterial density constructed using hyperbolic secant increases with surface tension at t = 0.1. $\tau _x = \tau _y = 10^2, k_x = k_y = 0.05$. (**a**) $D_2 = 1.62\cdot 10^{-3}$, (**b**) $D_2 = 16.2$, and (**c**) $D_2 = 162$. Other parameters as in Fig. 1.

**Figure 6:**
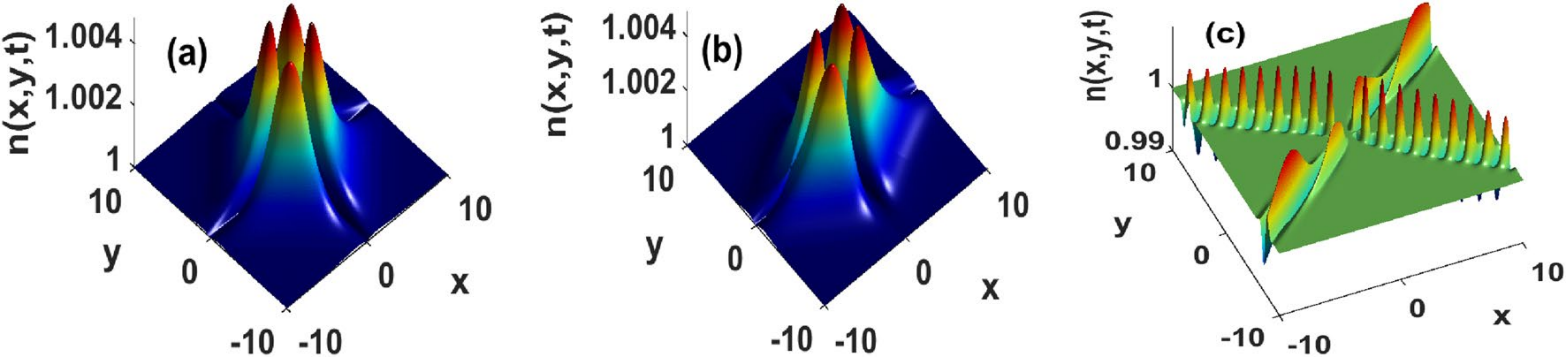
Strong incompressible flows significantly increase the amplitude approximated localized bacterial density determined using hyperbolic secant function at t = 0.1, and for $D_2 = 1.62$. (**a**): $\tau _x = \tau _y = 10^2$, (**b**) $\tau _x = \tau _y = 1$, and (**c**) $\tau _x = \tau _y = 10^{-2}$.

The sensitivity of the solutions Eq. (8) to surface tension and the strength of flows is displayed in Figs. 5 and  6, respectively. In presence of weak flows, the bacterial density increases with the strength of the surface tension Fig. 5. The initially maximally distributed density spreads towards the boundaries. Those spreads are akin to inverse diffusive fluxes in the sense they depopulate the position at which bacteria are initially deposited. If such a spreading remains well controlled, it may lead to phase-separated states reminiscent of those discussed in^7,45,50^. In addition, the surface tension contributes to splitting those structures into several aperiodic patterns of non-uniformly distributed heights Fig. 5. The non-uniform repartition is accompanied by an amplitude increase, and we suspect such coupled dynamics to be the signature of an operating unstable process actively taking place in the medium. A quasi-similar conclusion can be drawn from Fig. 6 where increasing the strength of fluid flows leads to an increase in bacterial density and the generation of several breathing structures with non-uniform amplitudes. This means that the strength of flows and surface tension not only facilitate bacterial dispersion but can also be used to redistribute bacteria at several locations across the domain. In groundwater treatment, those aggregations can be used to accelerate the degradation of a chemoattractant contaminant located at a specific position. Similar results were obtained in heterogeneously stratified media^37–39,42^. Our findings emphasize that surface tension, and strong fluid flows may provide bacteria with the extra energy that can allow them to travel faster, and carry more bacteria laying along their path. This is similar to results obtained^39^. Although their model did not account for surface tension nor bacterial proliferation, the authors showed that flows can enhance the transverse migration for chemotactic bacteria in porous media. Thus surface tension and the strength of flows within which cells are immersed significantly contribute to increasing the number of cells transported, a result that is on line with^51^. The latter showed that flows-induced density increase enhances the fertilization probability of the algae Volvox. Our study complements those findings by indicating that such a transport mode exhibits fast-spreading features. In order for cells to locally accommodate such a fast-spreading, their response time to external factors must significantly reduce. This implies several modifications of biochemical processes operating within cells. In other words, by carefully manipulating surface tension and the fluid flows, one can tune the internal biochemistry of cells such that their response to environmental changes improves or worsens.

**Figure 7:**
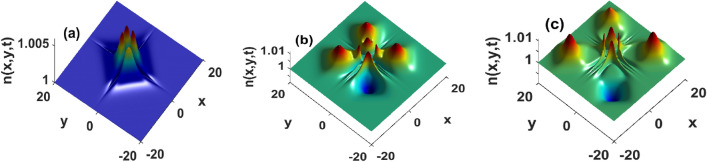
Diffusion initiates a fast scattering of initially localized bacterial density across the domain t = 0.1, and for $D_2 = 1.62$. (**a**): $\alpha = 1$, (**b**) $\alpha = 20$, and (**c**) $\alpha = 50$.

On the other hand, the augmentation of the number of cells transported at small flows indicates hydrodynamic interactions may not be the major factor of bacterial transport. The density increase at high flows suggests that the chemotactic activity of cells may not be strong enough to allow them to escape the advective flows imposed by the fluid. The latter observation enforces choosing with great care the nature of the fluid in real experimental situations. Figure 7 displays the influences the nonlinear diffusion has on bacterial density. In fact, higher values of $\alpha$ imply that the density-dependent diffusion decreases slowly hence entailing a fast diffusion rate. Our results thus indicate in addition to increasing the number of cells transported, such a rapid diffusion of cells entails a fast-spreading speed, hence emphasizing the idea of density expansion^19^. In groundwater treatment, such an observation is very promising because it indicates that in a porous medium where diffusion is sufficiently strong, the number of cells can be drained out rapidly. The higher values of $\alpha$ are used to obtain Fig. 7 implies that bacteria intrinsically diffuse faster than the chemoattractant. It is important to note that only the reverse has been observed during experiments in homogeneous media^1,29–31,41^. Hence from Fig. 7, we hypothesize that in order to generate fast propagating aggregations, the medium must be doped such that bacteria diffuse faster than chemicals in the medium.

## Conclusion

The investigations presented in this paper come into play when there is a great need to improve our understanding of collective transport in heterogeneous media. A two-dimensional chemotaxis model accounting for volume filling, surface tension, and non-uniformly imposed flows is considered. Through linear stability analysis, we show that the compressible/incompressible nature of the fluid deeply reshapes the stability and the domains where coherent patterns emerge. Two localized profiles, namely Gaussian and hyperbolic secant are used to probe the spatiotemporal evolution of weakly modulated nonlinear excitations. In dry media, Gaussian distributions travel faster, but secant ones exhibit better stability properties. In nonuniform fluids, the strength of the flows and the surface tension significantly reduce the global stability, hence pushing the system towards unstable regimes. Gaussian perturbations are shown to be the onset of concentric breathing patterns akin to propagating cyclic waves. Meanwhile, secant profiles give rise to non-uniformly distributed patterns of spots that slowly fill the domain in a quasi-symmetrical fashion. The results obtained using Gaussian and secant functions are significantly different and thus can be used to explain different physiological conditions. For instance, the fast spreading of Gaussian profiles may imply the latter strongly modulates the activity of bacteria while the slow spreading of secant may be a sign of low bacterial activity. Thus secant bacterial distributions are good candidates to monitor a slow progression of chemotactic cells in heterogeneous media.

## Supplementary Information

Supplementary Information.

## Data Availability

All data generated or analyzed during this study are included in this published article [and its supplementary information files].
