# Supplementary material for: Surface tension coupled non-uniformly imposed flows modulate the activity of reproducing chemotactic bacteria in porous media
Source: Sci Rep. 2023 Apr 5;13:5561. doi: 10.1038/s41598-023-31753-y (PMC10076314; doi:10.1038/s41598-023-31753-y)
Supplement: Supplementary file 1 — Supplementary Information. [file 41598_2023_31753_MOESM1_ESM.pdf]

Surface tension coupled non-uniformly imposed flows modulate the activity of reproducing chemotactic bacteria in porous media

W. Kuipou[1,2]*, A. Mohamadou[3]

[1] African Centre for Advanced Studies, P.O. Box 4477, Yaounde, Cameroon

[2] Centre for Research in Infectious Diseases, P.O. Box 13591, Yaounde, Cameroon

[3] National Advanced School of Engineering of Maroua, University of Maroua I, P.O. Box 46, Maroua, Cameroon

April 4, 2023

Supplementary file

Appendix A

The parameters of Eqs. (2) are defined as follows:

$$
\begin{aligned}
&a_{12} = \nabla^2 p(\mathbf{r},t) + |\nabla p(\mathbf{r},t)|^2, \quad a_{11} = D_2\left[\nabla^4 p + |\nabla p|^4 + 4\nabla p\cdot\nabla^3 p + 6\nabla^2 p|\nabla p|^2 + 3\left(\nabla^2 p\right)^2\right] - \alpha D_0 a_{12} - F_n \\
&\quad + 2D_2\left[\frac{\partial^2 p}{\partial x^2}\frac{\partial^2 p}{\partial y^2} + \frac{\partial^2 p}{\partial x^2}\left(\frac{\partial p}{\partial y}\right)^2 + \frac{\partial^2 p}{\partial y^2}\left(\frac{\partial p}{\partial x}\right)^2 + \left(\frac{\partial^2 p}{\partial x\partial y}\right)^2 + 2\frac{\partial p}{\partial x}\frac{\partial p}{\partial y}\frac{\partial^2 p}{\partial x\partial y}\right], \\
&a_{22} = -G_c - a_{12}, \quad \Delta = (a_{11}-a_{22})^2 + 4G_n\left(F_c - \chi_1 a_{12}\right), \quad D_0 = (1-\Phi n_0)\,e^{-2\Phi n_0}, \quad \chi_1 = \frac{\chi_0 n_0}{(1+c_0)^2}, \\
&F_n = \frac{\partial f}{\partial n}|_{n=n_0,c=c_0}, \quad F_c = \frac{\partial f}{\partial c}|_{n=n_0,c=c_0}, \quad G_n = \frac{\partial g}{\partial n}|_{n=n_0,c=c_0}, \quad G_c = \frac{\partial g}{\partial c}|_{n=n_0,c=c_0},
\end{aligned} \tag{A.1}
$$

*To whom correspondences should be addressed: kuipouwilliam@acas-yde.org
